# Comments on the Recent Address of Dr. Allport, before the Boston Academy of Dental Science

**Published:** 1875-03

**Authors:** J. Richardson

**Affiliations:** Terre Haute, Ind.


### SELECTED ARTICLES.

### ARTICLE V.

### *Comments on the Recent Address of Dr. Allport before the Academy of Dental Science, Boston, Mass.*

BY J. RICHARDSON, TERRE HAUTE, IND.

We have been interested in the perusal of the above address, partly because it is from the pen of a well known and accomplished practitioner whose views generally command attention, but chiefly because it affords a convenient text for the consideration of a question which has not been heretofore extensively discussed, namely, the status of our profession in its relation to medical science.

The attentive reader of this address will not fail to notice that the scheme of separating mechanical from medical, surgical, and operative practice, has its birth in an overweening ambition to ultimately secure from the medical faculty a full and unqualified recognition of our calling as a medical specialty. All this talk about the divorcement of these departments of dental practice is intended but as a means to this end,—a tub thrown to the whale,—a sort of propitiatory or sacrificial offering up of the professional rabble to secure a prospective and everlasting inheritance of glory for the elect few. Indeed, we are left in no doubt about this ulterior purpose. The doctor explicitly says:

"With the development of this higher mission of our profession there will be no occasion for the spectacle of dentology, with the grimace and shuffle of the mendicant, approaching the gates of the medical profession, and with downcast eyes begging a crumb of recognition. But with the accomplished separation of the two callings, heretofore combined in our practice, dentology, enriched by the experience of the special literature of the last half century, and the foundation of its practice laid exclusively in the science of medicine, rather than divded between that and a trade, the incongruity of the past will, in a few years disappear, and by deriving its nourishment from the body of which it is a branch, it will become more, and still more assimilated to the science and practice of medicine, and without demand, there will, both by the public and the medical faculty, be accorded, not to individual practitioners, but to the branch a full and cordial recognition as a specialty in medicine."

Thus, in imitation of the professional beggar, who, failing by other means to excite compassion and alms-giving, resorts to self-mutilation, the author of the address before us coolly proposes that the dental profession, which, by implication, he charges with presenting the spectacle of a mendicant at the door of the medical profession beseeching in vain for a crumb of recognition, shall now, as a forlorn hope, be thrust forward in all its self-imposed rags and shameless pretense of poverty in the superadded role of cripple. The doctor fairly chuckles over the shrewdness of his device, and rubs his hands together with unctious satisfaction in contemplation of the mollifying effect this unsightly deformity will have when added to the "grimace and shuffle" of his pet mendicant.

But, unfortunately for the author's designs, the plain fact is that he totally misapprehends the grounds on which the medical profession withholds recognition of our calling as a medical specialty. It makes no such demand as that implied by Dr. Allport. If this project of dismemberment were an accomplished fact to-day, he would not be so much as the breadth of a hair nearer the realization of his ambitious dream. Could he lop off at pleasure every branch of special practice having the slightest taint of mechanism about it,—the construction of artificial sets of teeth, obturators, appliances for dental irregularities, contrivances for fractures, the operation of filling teeth, the improvising of needed instruments and appliances for special or anomalous purposes, and so on, until the atmosphere of his office should become so purified that his professional olfactories should be no longer offended by the vile odors from the shop, but delight rather in the subtle and delicate aroma arising like grateful incense from pure and undefiled medical science, yet would he, despite all this trimming, and all his vaulting aspirations for higher medical culture, be still found waiting with "downcast eyes" in the attitude of a suppliant at the threshold of the medical profession with the same chances as now of being unceremoniously booted from the premises.

Dentistry, construed according to the standard of requirements prescribed by the medical profession from time immemorial in respect to other departments of the healing art, can be regarded as, in any proper sense, a medical specialty. Nor, we venture to affirm, will it ever be so accepted without a virtual abandonment of its distinctive organization as a professional body. The restless and unsatisfied spirits who are continually possessed with the mania for recognition will do well to accept the decision of the medical profession on this point as authoritatively enunciated lately by the editor of the Philadelphia *Medical Times* when he declares that, " If dentistry in the abstract is worthy of the position as a medical specialty, the living concrete dentistry can only gain such honor by a complete reorganization of the profession." Commenting upon the aids which the profession has derived from dental colleges, he remarks : " They ( dental colleges ) are an insuperable bar to its, (the profession) ever becoming a medical specialty, and the degree of D. D. S. is a badge of partial culture which must shut out from the medical ranks every one who wears no other insignia. * * * * So long as the dental profession by their deeds say, that such half culture is all that is necessary for a dentist, why should the members complain if the world and the *Times* agree with them and assign to dentistry the position which it at present holds." And again : " There is only one way by which a higher position can be achieved, and the first step is the abolition of the dental colleges and an enforcement of the idea that a general medical education must precede the special one."

Reorganization then is the condition precedent of recognition, and not the puerile deodorizing expedient dished up for us by Dr. Allport. If, in order to pass the sacred portals of the Aesculapian temple, we must needs suffer amputation of some objectionable part of the body professional, it is imperatively demanded that we shall offer the head rather than the extremities to the medical guillotine.

When we come to reflect how little identity there is between the dental profession and the recognized medical specialties, we shall feel less chagrined at the declaration of the *Times* editor that " the claim that dentistry is a branch or specialty of medicine is generally met by internal cachinations, whatever external behavior the laws of politeness enforce."

In what respects then does dentistry differ from the avowed medical specialties ?

1. A medical specialty proper, being the study and practice of a special department of general medicine, always pre-supposes an antecedent acquaintance with all the various branches of medical science. It is indeed but a creature, a product so to speak, of medical science, unrecognizable by any other relationship. This requirement of a general medical education therefore implies *whole* medical culture.

Now it is not claimed that any such general medical education is necessary in the case of the dental profession. The course of medical studies embraced in the curriculum of our dental colleges, and which may be taken as an expression of the highest medical attainment in our calling, does not by any means include every branch of the medical sciences, and therefore implies only *partial* medical culture.

In this view of the case, the lack of identity between dentistry and the medical specialties proper is apparent. It may be farther confined by an interpretation of the medical and dental degree. M. D. is the specialist's badge of medical culture, and signifying Doctor in Medicine, implies wholeness, or completeness. D. D. S. is the dentist's badge of medical culture, and signifying Doctor in Dental Surgery, which is but a *department* of medicine, plainly implies only something partial or fragmentary.

2. We style ourselves a *profession*. It is quite the fashion amongst us to boast of this, and there are none so tenacious of this characterization as those who clamor for recognition as a medical *specialty*. Now a profession, as designating any particular calling or avocation in life, implies unity, oneness, wholeness, completeness. Thus, amongst others, we have the medical and legal professions. Each of these has a distinctive educational system suitable to its organic structure, and commensurate with the requirements of all the multiform details of its peculiar work. However, many special departments may grow up within the body of these organizations to meet the exigencies of practice, still the idea of unity or wholeness must always attach to them in aggregated character of a profession. Dentistry is prop.

erly enough classified as a profession, having within itself all the necessary educational machinery to render the study and practice of all its departments complete. In the completeness and unity of its organic structure it is identical with the other professions mentioned. Here then is another point of departure in which identity is completely lost. By what process of legerdemain or hocus-pocus can it be a profession and a specialty at one and the same time? It would be very like a father claiming to be his own son, or a son his own father. If dentistry is properly a profession, it cannot also be a specialty, for one implies a whole and the other a part, and these plainly are not co-equal or identical except on the illogical assumption that the whole is equal to a part, or a part equal to the whole. If dentistry is a profession it can not be a specialty, for the whole can not be equal to a part, and if it is a specialty it can not be a profession, for a part can not be equal to the whole.

Surgery and opthalmology are specialties in medicine, but who ever heard of the surgical and opthalmological professions? In the legal profession the study and practice of commercial law is recognized as a legal specialty, but has it ever entered into the head of any one to designate this special practice as a profession? Operative and mechanical practice are regarded as specialties in dentistry, but no one has ever characterized them as professions.

Viewing the subject in this light, it seems incredible that any one of average intelligence should indulge the expectation that dentistry can ever become recognized as a specialty in medicine and yet retain its distinctive character of a profession. The proposition is not only absurd but presumptuous. If any one of the now recognized medical specialties were to take on such airs, it would be summarily ejected from the medical profession.

3. But perhaps the most conclusive reason why dentistry can not be recognized as belonging to the family of medical specialties, is because there is no blood relationship. Recognition is the birth-right of the avowed medical specialties, for they are legitimate offspring begotten within the body of the medical profession, and unchallenged heirs to all the rights, dignities, and immunities conferred by the doctorate in medicine. But dentistry is a body wholly alien to the medical profession by reason of its extraneous origin. It was born, so to speak, outside of wedlock, and is therefore barred from all rights of inheritance. It never had any acknowledged parentage, and is not even positively known to have been born at all, but, like Topsy, it probably "just grow'd." It is probably therefore "nobody's child." But it has thrived wonderfully nevertheless through all the period of its growth and development, counting from the time when it was, in some mysterious manner "ushered into this breathing world but half made up, and that so lame and unfashionable that the very dogs barked at it" to the present, when it may fairly challenge the admiration of mankind for its marvelous comeliness of form and feature. And this is the symmetrically-built, well-favoured, self-assuming, "child of fortune" whose fair proportions our distinguished friend proposes to mar for a consideration somewhat less substantial than a "mess of pottage."

But our medical friends will not compound on such terms. They are inexorable in their demand that we *shall be born again*, which means that, as a distinctive profession, we shall first suffer disorganization and death, and then become reorganized and legitimatized within the body of the medical profession by the usual processes of conception, utero-gestation and accouchment.

To this complexion must it come at last, if we are to be counted in as a medical specialty. Who in our profession are willing to abandon our present organization for the doubtful honor of being tacked on as a fragmentary part of the medical profession and dubbed medical specialists. If there are any such, let them speak and they shall have a respectful hearing. But until we are prepared for such a step let us be spared all this twaddle about the recognition our calling as a medical specialty.

It was no part of our original purpose to discuss in this article the scheme of separating the mechanical from the other departments of dental practice proposed by Dr. Allport. It certainly is sufficiently vulnerable to invite criticism, and we may address ourself to that particular feature of his address at another time.—*Dental Register.*

